# A miRNA-Driven Inference Model to Construct Potential Drug-Disease Associations for Drug Repositioning

**DOI:** 10.1155/2015/406463

**Published:** 2015-02-19

**Authors:** Hailin Chen, Zuping Zhang

**Affiliations:** ^1^School of Software, East China Jiaotong University, Nanchang 330013, China; ^2^Intelligent Optimization & Information Processing Lab, East China Jiaotong University, Nanchang 330013, China; ^3^School of Information Science and Engineering, Central South University, Changsha 410083, China

## Abstract

Increasing evidence discovered that the inappropriate expression of microRNAs (miRNAs) will lead to many kinds of complex diseases and drugs can regulate the expression level of miRNAs. Therefore human diseases may be treated by targeting some specific miRNAs with drugs, which provides a new perspective for drug repositioning. However, few studies have attempted to computationally predict associations between drugs and diseases via miRNAs for drug repositioning. In this paper, we developed an inference model to achieve this aim by combining experimentally supported drug-miRNA associations and miRNA-disease associations with the assumption that drugs will form associations with diseases when they share some significant miRNA partners. Experimental results showed excellent performance of our model. Case studies demonstrated that some of the strongly predicted drug-disease associations can be confirmed by the publicly accessible database CTD (www.ctdbase.org), which indicated the usefulness of our inference model. Moreover, candidate miRNAs as molecular hypotheses underpinning the associations were listed to guide future experiments. The predicted results were released for further studies. We expect that this study will provide help in our understanding of drug-disease association prediction and in the roles of miRNAs in drug repositioning.

## 1. Introduction

Over the past decades, the dominant assumption on drug research and development (R&D) was to design exquisitely selective ligands that act on a single disease target. However, such “one drug, one gene, one disease” paradigm was challenged in many studies and the concept of polypharmacology [1, 2] has later been proposed for those drugs acting on multiple targets rather than one target. The polypharmacological feature lays a solid foundation for the strategy of drug repositioning [3] which is to find new therapeutic uses for existing or failed drugs. One successful example is sildenafil which was first developed to treat angina but whose another effect of prolonged penile erections in human volunteers led to a new therapeutic indication for the drug. Moreover, the valuable safety information on existing drugs, like safety profiles and pharmacokinetic profiles, may greatly reduce the costs and risks associated with the early development stage and shorten routes to approval for therapeutic indications [4]. Therefore, drug repositioning has attracted enormous research interest from both academia and pharmaceutical companies.

Traditional drug repositioning efforts have spanned the spectrum from blind screening methods of chemical libraries against specific cell lines [5] or cellular organisms [6, 7], to serial testing of animal models [8]. As more and more biomolecular information is available, many computational strategies have been proposed for drug repositioning. More details are available at review [9]. These computational methods can be categorized into either drug-based approaches or disease-based approaches [9]. Drug-based computational methods utilize knowledge of chemical similarity [10–12], molecular activity similarity [13–15], or molecular docking [16, 17] to search new indications for drugs. Disease-based computational methods apply information of associative indication transfer [18] and shared molecular pathology [19, 20] or side effect similarity [21] to enable the discovery of new uses for drugs.

More recently, many studies demonstrated that drugs can regulate microRNA (miRNAs) expression. For example, the expression levels of 32 miRNAs were changed after the treatment of trichostatin A in human breast cancer cell lines by microarray [22]. miRNAs are short (~22 nucleotides) regulatory RNAs that downregulate gene expression at the posttranscriptional level by inhibiting translation or initiating mRNA degradation [23, 24]. More and more evidences have shown that miRNAs play critical roles in many important biological processes, such as tissue development [25], cell growth [26], and cellular signalling [27]. Therefore, the inappropriate expression of miRNAs could lead to a broad spectrum of diseases, including cancer [26] and cardiovascular diseases [28]. Meanwhile, miRNAs have several attractive features, including specific secondary structures and conserved sequences, to be druggable [29], and miRNA therapeutics may be superior to a mixture of small interfering RNAs (siRNAs) that are specifically designed to reduce the expression of a given number of target genes [30]. As a result, specific miRNAs will be treatment targets for majority of diseases [29, 31, 32] and targeting miRNAs with drugs will provide a new type of therapy for human diseases [33], which have given rise to the field of miRNA pharmacogenomics [34]. One example of miRNA therapeutics is MRX34, the first cancer-targeted miRNA drug, which entered Phase I clinical trials in patients with advanced hepatocellular carcinoma in 2013 [30].

To sum up, miRNAs play important roles in drug development and disease treatment. However, little computational research on miRNA-based drug repositioning has been conducted. Therefore, developing computational methods, by integrating information on drugs, miRNAs and diseases, to predict potential drug-disease associations for drug repositioning is greatly needed. In this paper, we proposed a miRNA-driven computational model to predict associations between drugs and diseases for drug repositioning. In this model, experimentally confirmed drug-miRNA associations and miRNA-disease associations were combined. The hypergeometric test was next executed for each drug-disease pair to measure whether they significantly shared some miRNA partners and only these associations with a small *P* value (<0.05) were chosen as predicted drug-disease associations for further drug repositioning. Excellent performance could be received from our experimental results and case studies on four drugs demonstrated that some strongly predicted drug-disease associations can be supported by previous research, which showed the practical value of our model. It is expected that our inference model will facilitate research on drug repositioning.

## 2. Materials and Methods

### 2.1. Datasets

In this paper, the following two datasets were integrated in our inference model to predict drug-disease associations for drug repositioning. Here below we provided a brief description.

#### 2.1.1. The Drug-miRNA Associations

We downloaded the drug-miRNA associations from the SM2miR database [29] which curated the experimentally validated drugs' effects on miRNA expression in 20 species from published papers. After deleting the duplicate records and restricting the species to* Homo sapiens*, we finally received 2307 distinct drug-miRNA associations, including 161 drugs and 748 miRNAs.

#### 2.1.2. The miRNA-Disease Associations

The miRNA-disease association data used in our paper were downloaded from HMDD [35] whose experimentally confirmed associations between miRNAs and diseases were manually retrieved from literature. We obtained 5075 miRNA-disease associations consisting of 502 miRNAs and 396 diseases from the HMDD database.

### 2.2. Method Description

The basic principle of our inference model is as follows: Because the inappropriate expression of miRNAs will lead to many kinds of complex diseases and drugs can regulate the expression of miRNAs, it is logical to infer that a drug has a possibility of forming an association with a disease (an opportunity of drug repositioning) when they share some significant miRNA partners.

**Figure 1:**
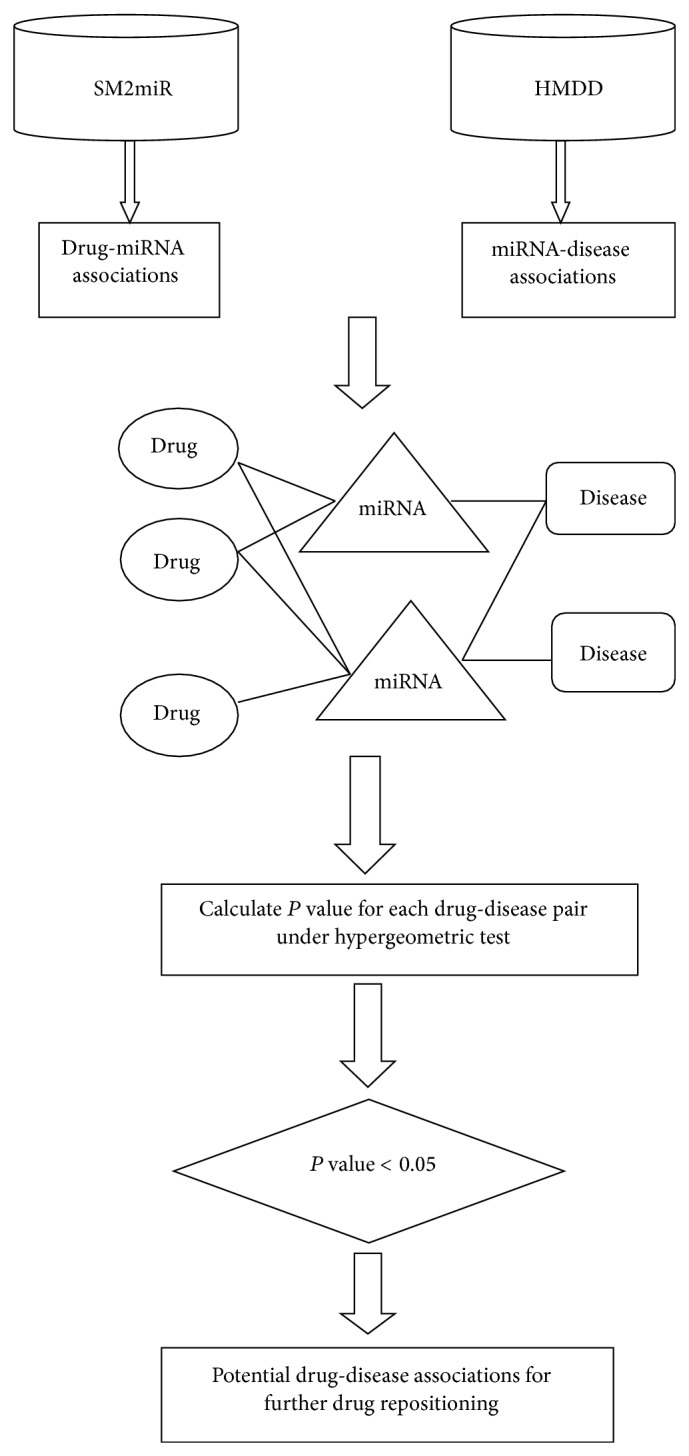
Principle and workflow of our inference model. The four steps of our model: (1) drug-miRNA association and miRNA-disease association extraction; (2) combination of the two sets of associations; (3) *P* value calculation; and (4) extraction of the significant overlaps of pairs between drugs and diseases.

To predict the associations between drugs and diseases, first experimentally verified drug-miRNA and miRNA-disease associations were combined. Next, for each drug-disease pair the hypergeometric test was executed separately to measure whether the drug and the disease significantly shared some miRNA partners which can interact with both of them. A *P* value was calculated as follows:

(1) $$\begin{array}{c} P=1-\overset{x-1}{\underset{i=0}{\sum }}\frac{\left(\begin{array}{c} L\\ i \end{array}\right)\left(\begin{array}{c} N-L\\ M-i \end{array}\right)}{\left(\begin{array}{c} N\\ M \end{array}\right)}, \end{array}$$

where *N* is the total number of miRNAs interacting with drugs or diseases, *M* is the number of miRNAs which interact with a given drug, *L* is the number of miRNAs interacting with a given disease. and *x* is the number of miRNAs that interact with both of them. Only the drug-disease pairs with a small *P* value (<0.05) were chosen as potential associations between drugs and diseases. The workflow of our inference model was illustrated in Figure 1.

## 3. Results

### 3.1. Construction and Analysis of Drug-miRNA Association Network and miRNA-Disease Association Network

In this study, we first focused on the experimentally supported drug-miRNA associations and miRNA-disease associations. The two sets of 2307 drug-miRNA associations and 5075 miRNA-disease associations were combined to infer potential drug-disease associations.

**Figure 2:**
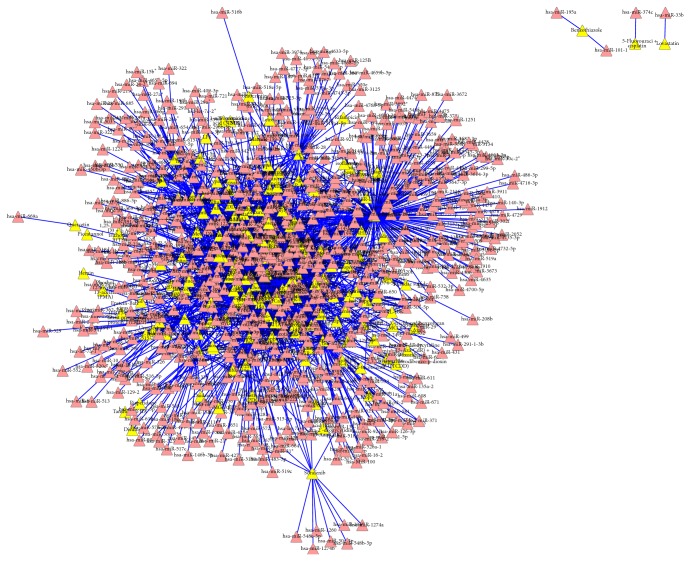
A global view of the drug-miRNA association network. The yellow triangles correspond to drugs and the red triangles correspond to miRNAs. An edge is drawn between a drug node and a miRNA node if there exists an experimentally supported association between the two nodes.

**Figure 3:**
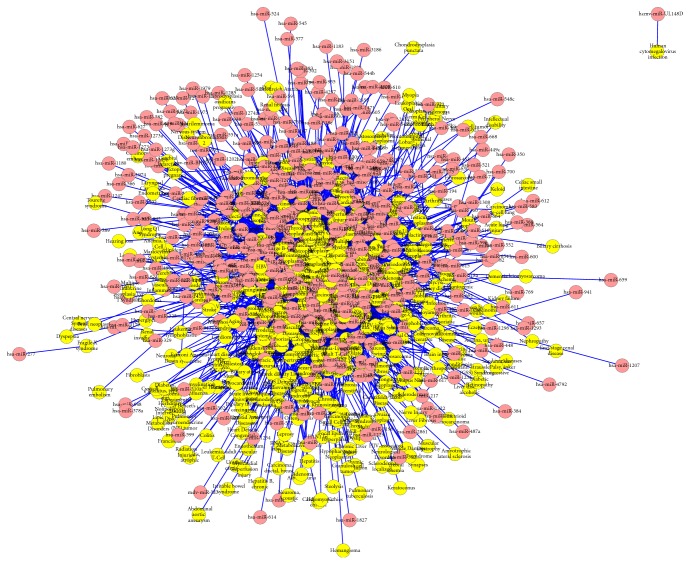
A global view of the miRNA-disease association network. The yellow circles correspond to diseases and the red circles correspond to miRNAs. An edge is drawn between a disease node and a miRNA node if there exists an experimentally supported association between the two nodes.

**Figure 4:**
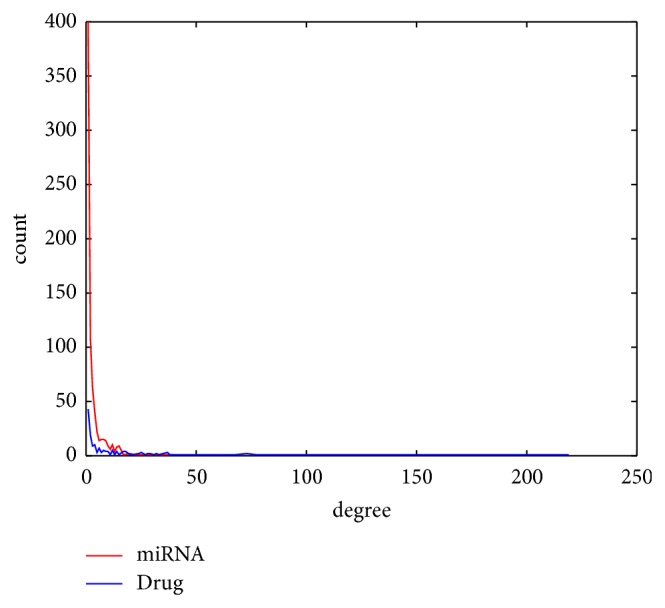
Degree distribution of drugs and miRNAs in the drug-miRNA association network.

**Figure 5:**
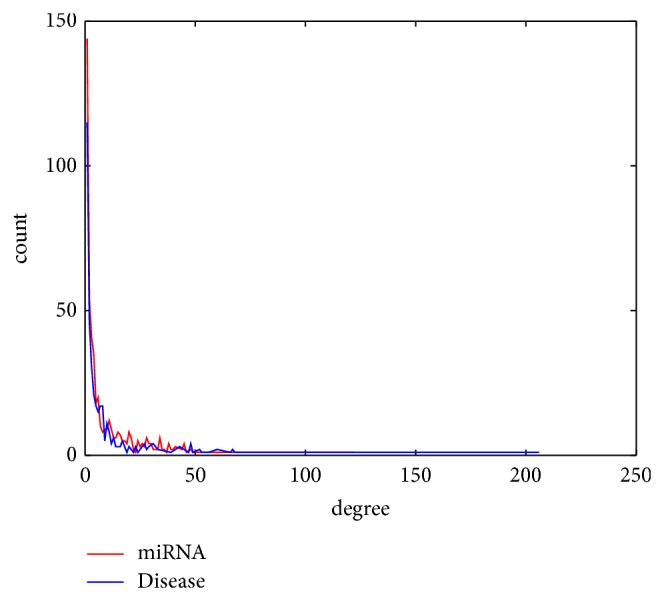
Degree distribution of diseases and miRNAs in the miRNA-disease association network.

We constructed the drug-miRNA association network and miRNA-disease association network using bipartite graph representation (see Figures 2 and 3, resp.) and analyzed some statistics for the two association networks (see Tables 1 and 2, resp.). Degree distribution of the two association networks can be seen in Figures 4 and 5, respectively.

### 3.2. Prediction of Associations between Drugs and Diseases on a Large Scale for Drug Repositioning

In this section, we conducted a comprehensive prediction of drug-disease associations for drug repositioning according to the steps in our inference model. The associations between each of the 161 drugs and the 396 diseases in our datasets were examined and we extracted the predicted drug-disease associations whose *P* values were smaller than 0.05 as the final results. Finally we received 8523 predicted drug-disease associations (see Supplementary Material S1 available online at http://dx.doi.org/10.1155/2015/406463). Moreover, the commonly interacted miRNAs were listed, which could provide guidance for biomedical experiments.

### 3.3. Performance Evaluation

To evaluate the performance of our model, we chose the latest version of CTD [36] database, which curates both scientific literature supported and inferred chemical-disease interactions by professionals, for results confirmation. After careful checking, we discovered that in the 8523 predicted drug-disease associations there are 2859 associations whose drugs and diseases can both be searched in the CTD [36] database and 2149 out of the 2859 associations (see Supplementary Material S2) can be confirmed in the database. This high success ratio (75.2% = 2149/2859) indicates an excellent prediction performance of our model and these unconfirmed associations provide opportunities for drug repositioning.

**Figure 6:**
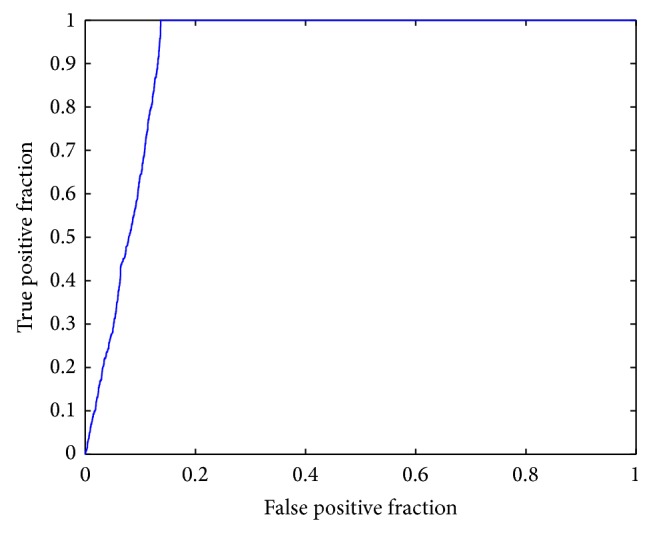
ROC curve by leave-one-out cross validation on the 448 experimentally supported associations.

Furthermore, we performed a leave-one-out cross validation (LOOCV) on experimentally verified drug-disease associations to test the inference ability of our model. Out of the 8523 predicted results, 448 literature-confirmed drug-disease associations were discovered. When the LOOCV procedure was implemented, each experimentally supported drug-disease association was left out as test association. Sensitivity and specificity for each threshold was calculated. A receiver-operating characteristics (ROC) curve (see Figure 6) was plotted by varying the thresholds, and a value of area under curve (AUC) of 0.9280 was received, which reinforced the power of our model to infer potential drug-disease associations for drug repositioning.

### 3.4. Case Studies

We chose four drugs (fluoxetine, bilobalide, docetaxel, and anthocyanin) as examples (see Tables 3–6) to demonstrate the effectiveness of our inference model. The predicted associations of the four drugs were further manually checked from the latest CTD [36] database. It could be observed that most of the prediction results (3/6, 9/26, 11/11, 2/3) have been confirmed. Since our current knowledge of drug-disease associations is incomplete, this validation results show excellent performance of our model. We can expect that the unconfirmed associations may also exist in reality.

We focused on the results of the drug fluoxetine for analysis. Fluoxetine (also known by the trade names Prozac, Sarafem, Ladose, and Fontex) is an antidepressant of the selective serotonin reuptake inhibitor (SSRI) class. In 2010, over 24.4 million prescriptions for generic formulations of fluoxetine were filled in the United States, making it the third most prescribed antidepressant after sertraline and citalopram (http://en.wikipedia.org/wiki/Fluoxetine). Therefore understanding its mechanism of action is of great importance for drug repositioning. Six predicted drug-disease associations were received for the drug fluoxetine, three of which were supported by the database CTD [36]. Furthermore, we discovered that hsa-miR-27b is an important mediating factor in forming the associations between diseases and fluoxetine [37–39], which offered testable molecular hypotheses for drug repositioning.

## 4. Discussion

As drug repositioning can provide valuable benefits to both pharmaceutical companies and human beings, it has increasingly gained a lot of research interest. Computational predictions of the most promising drug-disease associations are important ways for drug repositioning. Therein, we developed an inference model to predict potential drug-disease associations for further drug repositioning based on the logic assumption that there will exist an association between a drug and a disease when they have some commonly related miRNAs.

Experimental results have shown the good performance of our model, which can be attributed to two factors: the data quality and the workflow strategy. The data integrated in our predictive model were extracted from highly reliable databases [29, 35] and they are supported by published papers. Furthermore, the model we used in this study was well defined and has proven to be successful in previous research [40, 41]. Compared to previous methods [9], our model does not need to use known drug-disease associations. Another advantage lied in our model is that testable hypotheses are provided and they can be used to guide further biomedical experiments in drug repositioning. Despite the encouraging results, it should be noted that the results produced by our model may be biased because current data of drug-miRNA and miRNA-disease associations are not complete. Therefore the performance of our model could be further improved by integrating more verified drug-miRNA and miRNA-disease associations.

## 5. Conclusion

In this paper, we proposed a computational model to predict potential drug-disease associations for future drug repositioning by considering commonly related miRNA partners. To the best of our knowledge, this is the first attempt to infer drug-disease associations, in which miRNAs that are likely to mediate the associations are explicitly included. Case studies on real drugs demonstrated the power of our model in drug repositioning. In total, our study reveals a promising perspective to study drug and disease relationships and to search new opportunities for drug repositioning.

## Supplementary Material

Supplementary Material S1: The 8523 potential drug-disease associations predicted by our inference model
Supplementary Material S2: The 2149 drug-disease associations confirmed by the CTD database

## Figures and Tables

**Table 1:** Statistics of the drug-miRNA association network.

Number of drugs	Number of miRNAs	Number of drug-miRNA associations	Average degree of drugs	Average degree of miRNAs	Sparsity

161	748	2307	14.3	3.1	0.019

**Table 2:** Statistics of the miRNA-disease association network.

Number of miRNAs	Number of diseases	Number of miRNA-disease associations	Average degree of miRNAs	Average degree of diseases	Sparsity

502	396	5075	10.1	12.8	0.026

**Table 3:** Prediction results of associated diseases for the drug fluoxetine.

Drug	Common miRNA(s)	Disease	*P* value	Evidence
Fluoxetine	hsa-miR-27b	Cardiomyopathy, hypertrophic	0.0325	CTD confirmed
Fluoxetine	hsa-miR-27b	Carcinoma, oral	0.0368	CTD confirmed
Fluoxetine	hsa-miR-27b	Osteoarthritis	0.0152	CTD confirmed
Fluoxetine	hsa-miR-27b	Oral lichen planus	0.00872	
Fluoxetine	hsa-miR-27b	Cryptosporidium	0.00872	
Fluoxetine	hsa-miR-27b	Dyslipidemia	0.00218	

**Table 4:** Prediction results of associated diseases for the drug bilobalide.

Drug	Common miRNA(s)	Disease	*P* value	Evidence
Bilobalide	hsa-miR-148a hsa-miR-27a hsa-miR-27b hsa-miR-328 hsa-miR-451 hsa-miR-519c	Breast neoplasms	5.51*E* − 04	CTD confirmed
Bilobalide	hsa-miR-27a hsa-miR-27b hsa-miR-328 hsa-miR-451	Carcinoma, non-small-cell lung	0.00178	CTD confirmed
Bilobalide	hsa-miR-148a hsa-miR-27b hsa-miR-328 hsa-miR-451	Colorectal neoplasms	0.00908	CTD confirmed
Bilobalide	hsa-miR-148a hsa-miR-27a hsa-miR-27b	Endometrial neoplasms	0.00408	
Bilobalide	hsa-miR-148a hsa-miR-27a hsa-miR-27b hsa-miR-328 hsa-miR-451 hsa-miR-519c	Gastric neoplasms	3.61*E* − 05	
Bilobalide	hsa-miR-148a hsa-miR-451	Gastrointestinal neoplasms	0.0149	
Bilobalide	hsa-miR-148a hsa-miR-27b hsa-miR-451	Glioblastoma	0.0212	
Bilobalide	hsa-miR-27a hsa-miR-451 hsa-miR-519c	Neoplasms	0.0294	CTD confirmed
Bilobalide	hsa-miR-148a hsa-miR-27a hsa-miR-451	Pancreatic neoplasms	0.0278	CTD confirmed
Bilobalide	hsa-miR-148a hsa-miR-27a hsa-miR-27b	Prostatic neoplasms	0.0286	CTD confirmed
Bilobalide	hsa-miR-148a hsa-miR-27a hsa-miR-27b	Carcinoma, squamous cell	0.0106	CTD confirmed
Bilobalide	hsa-miR-148a hsa-miR-27a hsa-miR-27b	Adenoviridae infections	0.0110	
Bilobalide	hsa-miR-27a hsa-miR-27b hsa-miR-451	Glioma	0.00433	
Bilobalide	hsa-miR-328 hsa-miR-451	Myocardial infarction	0.0184	CTD confirmed
Bilobalide	hsa-miR-148a hsa-miR-27b	Leukemia, lymphocytic, chronic, B-cell	0.0389	
Bilobalide	hsa-miR-451	Leukemia, myelogenous, chronic	0.0228	
Bilobalide	hsa-miR-27b	Oral lichen planus	0.0303	
Bilobalide	hsa-miR-148a	Amyloidosis	0.0228	
Bilobalide	hsa-miR-148a	Fibrodysplasia ossificans progressiva	0.00764	
Bilobalide	hsa-miR-27a	Lymphoma, extranodal NK-T-Cell	0.0152	CTD confirmed
Bilobalide	hsa-miR-27b	Cryptosporidium	0.0303	
Bilobalide	hsa-miR-27a	Heart diseases	0.0303	
Bilobalide	hsa-miR-27b	Dyslipidemia	0.00764	
Bilobalide	hsa-miR-328	Myopia	0.00764	
Bilobalide	hsa-miR-451	Erythropoiesis	0.00764	
Bilobalide	hsa-miR-451	Leukemia, myelogenous, chronic, BCR-ABL positive	0.00764	

**Table 5:** Prediction results of associated diseases for the drug docetaxel.

Drug	Common miRNA(s)	Disease	*P* value	Evidence
Docetaxel	hsa-miR-100 hsa-miR-200b hsa-miR-99b	Endometriosis	0.00629	CTD confirmed
Docetaxel	hsa-miR-100 hsa-miR-200b hsa-miR-502 hsa-miR-99b	Ovarian neoplasms	0.00850	CTD confirmed
Docetaxel	hsa-miR-100 hsa-miR-200b hsa-miR-99b	Prostatic neoplasms	0.0423	CTD confirmed
Docetaxel	hsa-miR-99b	Sarcoma, synovial	0.0260	CTD confirmed
Docetaxel	hsa-miR-100 hsa-miR-200b hsa-miR-99b	Urinary bladder neoplasms	0.0154	CTD confirmed
Docetaxel	hsa-miR-100 hsa-miR-200b	Adrenal cortex neoplasms	0.00182	CTD confirmed
Docetaxel	hsa-miR-100 hsa-miR-99b	Atherosclerosis	0.0129	CTD confirmed
Docetaxel	hsa-miR-100 hsa-miR-502	Muscular dystrophies	0.00290	CTD confirmed
Docetaxel	hsa-miR-200b	Glomerulonephritis, IGA	0.0430	CTD confirmed
Docetaxel	hsa-miR-200b	Tongue neoplasms	0.0260	CTD confirmed
Docetaxel	hsa-miR-200b	Diabetic retinopathy	0.00873	CTD confirmed

**Table 6:** Prediction results of associated diseases for the drug anthocyanin.

Drug	Common miRNA(s)	Disease	*P* value	Evidence
Anthocyanin	hsa-miR-429 hsa-miR-486	Barrett esophagus	0.0372	CTD confirmed
Anthocyanin	hsa-miR-1	Arrhythmias, cardiac	0.0389	CTD confirmed
Anthocyanin	hsa-miR-1	Chordoma	0.0197	

## References

[1] Hopkins A. L. (2008). Network pharmacology: the next paradigm in drug discovery. *Nature Chemical Biology*.

[2] Xie L., Xie L., Kinnings S. L., Bourne P. E. (2012). Novel computational approaches to polypharmacology as a means to define responses to individual drugs. *Annual Review of Pharmacology and Toxicology*.

[3] Ashburn T. T., Thor K. B. (2004). Drug repositioning: identifying and developing new uses for existing drugs. *Nature Reviews Drug Discovery*.

[4] Wu Z., Wang Y., Chen L. (2013). Network-based drug repositioning. *Molecular BioSystems*.

[5] Weinstein J. N., Myers T. G., O'Connor P. M. (1997). An information-intensive approach to the molecular pharmacology of cancer. *Science*.

[6] Hughes T., Andrews B., Boone C. (2004). Old drugs, new tricks: using genetically sensitized yeast to reveal drug targets. *Cell*.

[7] Lum P. Y., Armour C. D., Stepaniants S. B. (2004). Discovering modes of action for therapeutic compounds using a genome-wide screen of yeast heterozygotes. *Cell*.

[8] Kinnamon K. E., Rothe W. E. (1975). Biological screening in the U.S. Army antimalarial drug development program. *The American Journal of Tropical Medicine and Hygiene*.

[9] Dudley J. T., Deshpande T., Butte A. J. (2011). Exploiting drug-disease relationships for computational drug repositioning. *Briefings in Bioinformatics*.

[10] Keiser M. J., Setola V., Irwin J. J. (2009). Predicting new molecular targets for known drugs. *Nature*.

[11] Eckert H., Bajorath J. (2007). Molecular similarity analysis in virtual screening: foundations, limitations and novel approaches. *Drug Discovery Today*.

[12] Noeske T., Sasse B. C., Stark H., Parsons C. G., Weil T., Schneider G. (2006). Predicting compound selectivity by self-organizing maps: cross-activities of metabotropic glutamate receptor antagonists. *ChemMedChem*.

[13] Lamb J., Crawford E. D., Peck D. (2006). The connectivity map: using gene-expression signatures to connect small molecules, genes, and disease. *Science*.

[14] Iorio F., Bosotti R., Scacheri E. (2010). Discovery of drug mode of action and drug repositioning from transcriptional responses. *Proceedings of the National Academy of Sciences of the United States of America*.

[15] Wei G., Twomey D., Lamb J. (2006). Gene expression-based chemical genomics identifies rapamycin as a modulator of MCL1 and glucocorticoid resistance. *Cancer Cell*.

[16] Zahler S., Tietze S., Totzke F. (2007). Inverse in silico screening for identification of kinase inhibitor targets. *Chemistry & Biology*.

[17] Kinnings S. L., Liu N., Buchmeier N., Tonge P. J., Xie L., Bourne P. E. (2009). Drug discovery using chemical systems biology: repositioning the safe medicine Comtan to treat multi-drug and extensively drug resistant tuberculosis. *PLoS Computational Biology*.

[18] Chiang A. P., Butte A. J. (2009). Systematic evaluation of drug-disease relationships to identify leads for novel drug uses. *Clinical Pharmacology and Therapeutics*.

[19] Hu G., Agarwal P. (2009). Human disease-drug network based on genomic expression profiles. *PLoS ONE*.

[20] Suthram S., Dudley J. T., Chiang A. P., Chen R., Hastie T. J., Butte A. J. (2010). Network-based elucidation of human disease similarities reveals common functional modules enriched for pluripotent drug targets. *PLoS Computational Biology*.

[21] Campillos M., Kuhn M., Gavin A.-C., Jensen L. J., Bork P. (2008). Drug target identification using side-effect similarity. *Science*.

[22] Rhodes L. V., Nitschke A. M., Segar H. C. (2012). The histone deacetylase inhibitor trichostatin A alters microRNA expression profiles in apoptosis-resistant breast cancer cells. *Oncology Reports*.

[23] Bartel D. P. (2004). MicroRNAs: genomics, biogenesis, mechanism, and function. *Cell*.

[24] Filipowicz W., Jaskiewicz L., Kolb F. A., Pillai R. S. (2005). Post-transcriptional gene silencing by siRNAs and miRNAs. *Current Opinion in Structural Biology*.

[25] Krichevsky A. M., King K. S., Donahue C. P., Khrapko K., Kosik K. S. (2003). A microRNA array reveals extensive regulation of microRNAs during brain development. *RNA*.

[26] Esquela-Kerscher A., Slack F. J. (2006). Oncomirs—microRNAs with a role in cancer. *Nature Reviews Cancer*.

[27] Cui Q., Yu Z., Purisima E. O., Wang E. (2006). Principles of microRNA regulation of a human cellular signaling network. *Molecular Systems Biology*.

[28] Latronico M. V. G., Catalucci D., Condorelli G. (2007). Emerging role of microRNAs in cardiovascular biology. *Circulation Research*.

[29] Liu X., Wang S., Meng F. (2013). SM2miR: a database of the experimentally validated small molecules' effects on microRNA expression. *Bioinformatics*.

[30] Ling H., Fabbri M., Calin G. A. (2013). MicroRNAs and other non-coding RNAs as targets for anticancer drug development. *Nature Reviews Drug Discovery*.

[31] Qiu C., Chen G., Cui Q. (2012). Towards the understanding of microRNA and environmental factor interactions and their relationships to human diseases. *Scientific Reports*.

[32] Chen X., Liu M.-X., Cui Q.-H., Yan G.-Y. (2012). Prediction of disease-related interactions between microRNAs and environmental factors based on a semi-supervised classifier. *PLoS ONE*.

[33] Zhang S., Chen L., Jung E. J., Calin G. A. (2010). Targeting MicroRNAs with small molecules: from dream to reality. *Clinical Pharmacology and Therapeutics*.

[34] Shomron N. (2010). MicroRNAs and pharmacogenomics. *Pharmacogenomics*.

[35] Li Y., Qiu C., Tu J. (2014). HMDD v2.0: a database for experimentally supported human microRNA and disease associations. *Nucleic Acids Research*.

[36] Davis A. P., Grondin C. J., Lennon-Hopkins K. (2015). The comparative toxicogenomics database's 10th year anniversary: update 2015. *Nucleic Acids Research*.

[37] Rodrigues A. C., Li X., Radecki L. (2011). MicroRNA expression is differentially altered by xenobiotic drugs in different human cell lines. *Biopharmaceutics & Drug Disposition*.

[38] Akhtar N., Rasheed Z., Ramamurthy S., Anbazhagan A. N., Voss F. R., Haqqi T. M. (2010). MicroRNA-27b regulates the expression of matrix metalloproteinase 13 in human osteoarthritis chondrocytes. *Arthritis and Rheumatism*.

[39] Busk P. K., Cirera S. (2010). MicroRNA profiling in early hypertrophic growth of the left ventricle in rats. *Biochemical and Biophysical Research Communications*.

[40] Jiang Q., Hao Y., Wang G. (2010). Prioritization of disease microRNAs through a human phenome-microRNAome network. *BMC Systems Biology*.

[41] Liu M.-X., Chen X., Chen G., Cui Q.-H., Yan G.-Y. (2014). A computational framework to infer human disease-associated long noncoding RNAs. *PLoS ONE*.

